# Main causes of death in advanced biliary tract cancer

**DOI:** 10.1002/cam4.5794

**Published:** 2023-03-29

**Authors:** Kana Kimura‐Seto, Yasushi Kojima, Shiori Komori, Yuya Hisada, Yuki Otake, Yuka Yanai, Akiko Saito, Naoki Akazawa, Yasuo Tanaka, Chizu Yokoi, Mikio Yanase, Junichi Akiyama, Natsuyo Yamamoto, Kazuhiko Yamada

**Affiliations:** ^1^ Department of Gastroenterology National Center for Global Health and Medicine 1‐21‐1 Toyama, Shinjuku‐ku Tokyo 162‐8655 Japan; ^2^ Course of Advanced and Specialized Medicine Juntendo University School of Medicine Graduate School of Medicine 2‐1‐1 Hongo, Bunkyo‐ku Tokyo 113‐8421 Japan; ^3^ Department of Surgery National Center for Global Health and Medicine 1‐21‐1 Toyama, Shinjuku‐ku Tokyo 162‐8655 Japan

**Keywords:** chemotherapy, cholangiocarcinoma, clinical cancer research, prognosis, survival

## Abstract

**Background:**

There are no previous reports on the main causes of death in biliary tract cancer (BTC) patients. This study aimed to evaluate the main causes of death and survival rates in patients with BTC.

**Methods:**

We retrospectively evaluated 143 patients who were diagnosed with unresectable BTC between August 2010 and March 2020. We classified the main causes of death based on laboratory data, imaging studies, and medical records. The main causes of death evaluated included liver failure, cholangitis, cachexia, other causes associated with tumor progression, and complications. We also analyzed survival rates for each main cause of death.

**Results:**

After excluding patients who were lost to follow‐up, living patients, and patients who had no records of laboratory data within 30 days before the date of death, 108 patients were analyzed. The main cause of death was cholangitis in 33 (30.6%), cachexia in 22 (20.4%), liver failure in 10 (9.3%), other causes associated with tumor progression in 18 (16.7%), and complications in 25 (23.2%) patients. Median overall survival (OS) was 334.0 days in the chemotherapy group and 75.0 days in the best supportive care (BSC) group. Survival analyzed according to the main cause of death was significantly different between the chemotherapy and BSC groups; OS for cachexia, cholangitis, liver failure, other causes associated with tumor progression, and complications, respectively, were 453.0, 499.0, 567.0, 205.0, and 327.5 days (*p* = 0.003) in the chemotherapy group and 219.0, 69.0, 34.0, 93.0, and 56.0 days (*p* = 0.001) in the BSC group.

**Conclusion:**

The main causes of death in patients with advanced BTC are cholangitis, cachexia, liver failure, other causes associated with tumor progression, and complications. Other causes associated with tumor progression in the chemotherapy group, and liver failure in the BSC group as the main causes of death shortened the survival of BTC patients.

## Graphical abstract

The main causes of death in patients with advanced biliary tract cancer (BTC) are cholangitis, cachexia, liver failure, other causes associated with tumor progression, and complications. Other causes associated with tumor progression in the chemotherapy group, and liver failure in the BSC group as the main causes of death shortened the survival of BTC patients.

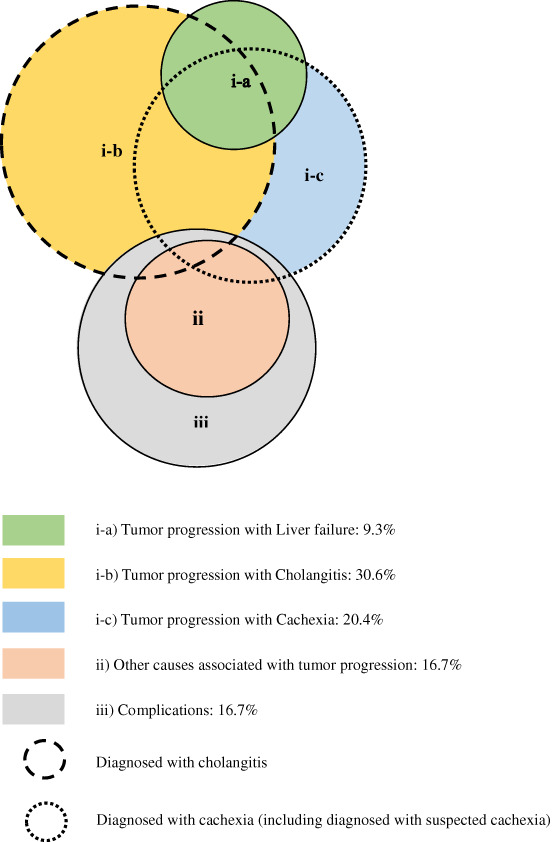

## 1 INTRODUCTION

Biliary tract cancer (BTC) involves cancer of various parts of the biliary tract, including the intrahepatic and perihilar bile duct, gall bladder, distal biliary tree, and ampulla of Vater.^1^ More than 20,000 patients are diagnosed with BTC in Japan annually, accounting for more than 2% of all cancers.^2^ The treatment of unresectable BTC is systemic chemotherapy consisting mainly of a combination of two or three drugs based on gemcitabine.^3^, ^4^, ^5^ However, despite developments in the treatment of unresectable BTC, the prognosis remains poor: BTC reportedly has the second‐worst survival rate after pancreatic cancer in Japan.^2^

Despite this, there are few previous reports on the main causes of death in BTC patients. A previous study examined the major causes of death in patients with intrahepatic cholangiocarcinoma, and broadly classified them into cancer and non‐cancer causes.^6^ However, that study included Stage Intrahepatic cholangiocarcinoma patients and did not examine the main cancer‐related causes of death. An old report from 1970 indicated the causes of death in cancer patients in general.^7^ In this study, we examined the main cancer‐related causes of death in BTC patients. Since we considered it important to examine the main cause of death clinically in order to predict the clinical course and prognosis of unresectable BTC, we examined the main cause of death, whether the main cause was due to cancer or something else, and assessed in detail the conditions associated with cancer as the main cause of death. Cachexia, liver failure, and cholangitis are known to contribute to the high mortality in cholangiocarcinoma.^8^, ^9^ Hence, we hypothesized that BTC might have disease‐specific causes of death. This study aimed to clarify the main causes of death in patients with unresectable BTC. We also investigated the possible influence of chemotherapy and the resultant adverse events on the main cause of death in unresectable BTC by stratifying the patients into the chemotherapy and best supportive care (BSC) groups.

## 2 METHOD

### 2.1 Study population and design

**FIGURE 1:**
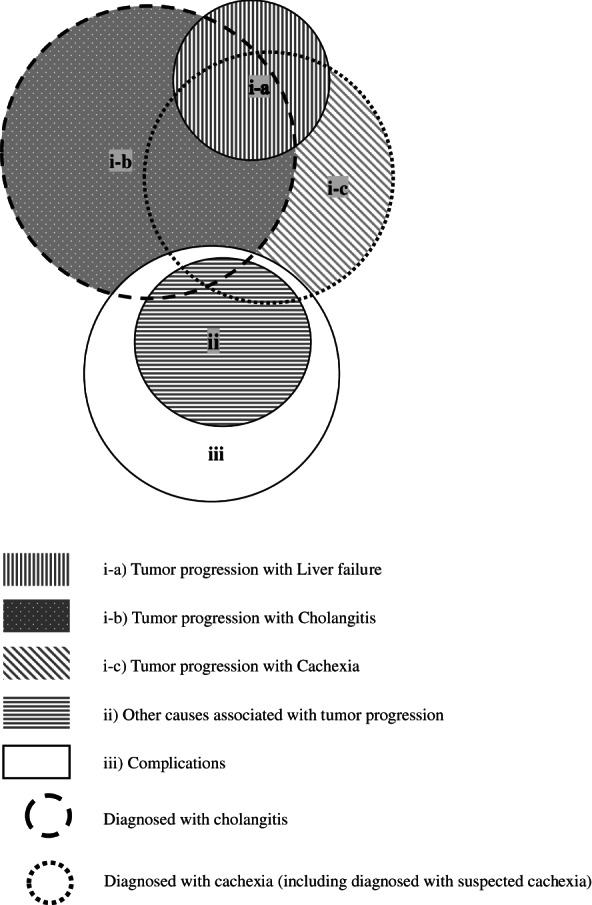
Main causes of death in cases classified as liver failure, cholangitis, cachexia, other causes associated with tumor progression, and complications. The main causes of death were classified as follows: (i) leading causes associated with tumor progression, such as (a) liver failure, (b) cholangitis, (c) cachexia; (ii) other causes associated with tumor progression; and (iii) complications. Cholangitis was defined as the main cause of death in patients who had cholangitis and in whom no other cause of death could be identified. Patients with cachexia as the main cause of death included those who were diagnosed with cachexia or “suspected cachexia,” and in whom other causes of death could not be diagnosed; additionally, these patients did not have cholangitis or liver failure.

We retrospectively analyzed unresectable BTC patients. This study was conducted from August 2010 to March 2020 at the National Center for Global Health and Medicine, Tokyo, Japan. We analyzed the main causes of death in BTC patients who died in September 30, 2021. The main causes of death were classified based on laboratory data within 30 days before the date of death, the most recent imaging studies and the patients' medical records as (i) leading causes associated with tumor progression, such as (a) liver failure, (b) cholangitis, (c) cachexia; (ii) other causes associated with tumor progression, such as metastasis; and (iii) complications, such as renal failure, heart failure, pneumonia, and complications of procedures, the criteria for which are defined below (Figure 1).

Liver failure was defined as the main cause of death in patients who met the following two criteria based on previous reports^10^, ^11^, ^12^: (A) tumors occupying more than 80% of the liver or more than 60% in cases with cirrhosis or suspected circulatory failure of the liver, and (B) suspected liver dysfunction based on a PT‐INR of >1.5 or coma with hyperammonemia.

Patients were defined as having cholangitis if they met the following three criteria according to the TG 18 of the Updated Tokyo Guidelines for the Management of Acute Cholangitis and Cholecystitis^13^: (i) laboratory data: WBC <4 or >10 × 1000/μL and CRP ≥1 mg/dL; (ii) jaundice (total bilirubin ≥2 mg/dL) or abnormal liver function tests (alkaline phosphatase (IU), γ‐glutamyl transpeptidase (IU), aspartate aminotransferase (IU) and alanine aminotransferase (IU) >1.5 times the upper limit of normal); and (iii) Imaging observation of biliary dilation. Cholangitis was considered as the main cause of death in patients who had cholangitis and in whom no other cause of death could be identified.

We diagnosed cachexia according to the European Palliative Care Research Collaborative (EPCRC) definition of cachexia in patients who met the following criteria: Weight loss >5% over the past 6 months (in the absence of simple starvation), or BMI <20 kg/m^2^ and any degree of weight loss >2%. Although the EPCRC diagnostic criteria include sarcopenia and any degree of weight loss >2%, no cases were diagnosed with this criterion in this study because appendicular skeletal muscle index measurements were unavailable.^14^ Patients whose weight was not well‐documented in their medical records and who were not diagnosed with cachexia according to the EPCRC diagnostic criteria but who met three of the following five criteria, as defined by Evans et al. were diagnosed with “suspected cachexia”: decreased muscle strength, fatigue, anorexia, low fat‐free mass index, and abnormal biochemistry (increased inflammatory markers [CRP], anemia [Hb <12 g/dL] or low serum albumin [<3.2 g/dL]).^15^ Since fat‐free mass index was not measured in this study, the diagnosis was based on the fulfillment of the four items except “low fat‐free mass index.” Patients with cachexia as the main cause of death included those who were diagnosed with cachexia or “suspected cachexia,” and in whom other causes of death could not be diagnosed from medical records or blood tests; additionally, these patients did not have cholangitis or liver failure.

For analyses and to assess the effects of treatment on study outcomes, the patients included in this study were divided into two groups: those who received chemotherapy (chemotherapy group) and those who did not receive chemotherapy (BSC group).

### 2.2 Statistical analysis

Survival curves and results were estimated using the Kaplan–Meier method and compared using the log‐rank test. Survival time was defined from the date of diagnosis to the date of death. Bonferroni's multiple comparison tests were used to compare survival between groups.

We used Mann–Whitney *U*‐tests to compare the medians of continuous variables (such as age) and Fisher's exact tests to compare the proportions of categorical variables (such as sex) between the groups. A *p*‐value of less than 0.05 was considered statistically significant. All statistical analyses were performed with EZR software (Saitama Medical Center, Jichi Medical University, Saitama, Japan), a graphical user interface for R (The R Foundation for Statistical Computing). More precisely, it is a modified version of R commander designed to add statistical functions frequently used in biostatistics.^16^

## 3 RESULTS

### 3.1 Demographic and clinical features

**FIGURE 2:**
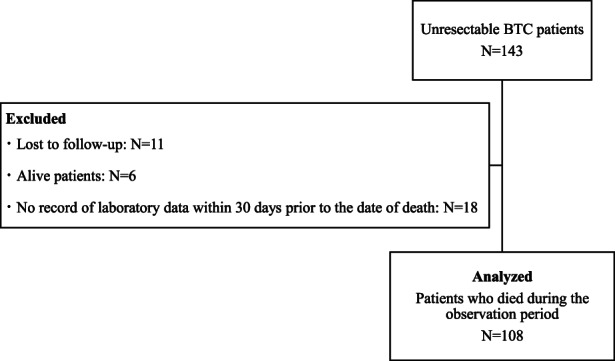
Flow diagram of patient selection. Of the 143 unresectable BTC patients who were eligible for study participation, 126 patients died during the observation period. Among these 126 patients, 18 patients were excluded because they had no record of laboratory data within 30 days before the date of death, and the remaining 108 patients were analyzed.

Of the 143 unresectable BTC patients who were eligible for study participation, 126 patients died during the observation period. Among these 126 patients, 18 patients were excluded because they had no record of laboratory data within 30 days before the date of death, and the remaining 108 patients were analyzed (Figure 2). Patient characteristics at the time of diagnosis of unresectable BTC are shown in Table 1. Median patient age was 75 years (range: 43–99). Of them, 52 patients (91.2%) in the chemotherapy group and 33 patients (64.7%) in the BSC group were Eastern Cooperative Oncology Group Performance Status **(**ECOG PS) 0 or 1. Regarding the primary tumor site, 29 patients (26.9%) had intrahepatic cholangiocarcinoma, 27 patients (25.0%) had perihilar cholangiocarcinoma, 36 patients (33.3%) had gallbladder carcinoma, 13 patients (12.0%) had distal biliary tree cholangiocarcinoma and 3 patients (2.8%) had ampullary carcinoma. In terms of pathological tumor type, 83 patients (76.9%) had adenocarcinoma, while tumor tissue was not available from 21 patients (19.4%). Fifty‐one patients (47.2%) had no other anti‐cancer treatment, and 20 patients (18.5%) underwent resection of the primary lesion before systemic chemotherapy.

**TABLE 1:** Characteristics of the patients at the time of diagnosis.

Variable	Chemotherapy group (*N* = 57)	BSC group **(** *N* = 51)	Total **(** *N* = 108)	*p*‐Value
Age, years, median (range)	69 (43–89)	80 (51–99)	75 (43–99)	0.0002
Sex, *n* (%)				
Females	13 (22.8)	17 (33.3)	30 (27.8)	0.28
Males	44 (77.2)	34 (66.7)	78 (72.2)	
ECOG PS, *n* (%)				
0	36 (63.2)	11 (21.6)	47 (43.5)	0.000001
1	16 (28.1)	22 (43.1)	38 (35.2)	
2	5 (8.8)	4 (7.8)	9 (8.3%)	
3	0	9 (17.7)	9 (8.3%)	
4	0	5 (9.8)	5 (4.6%)	
Primary tumor site, *n* (%)				
Intrahepatic	20 (35.1)	9 (17.7)	29 (26.9)	0.051
Perihilar	11 (19.3)	16 (31.4)	27 (25.0)	0.18
Gall bladder	19 (33.3)	17 (33.3)	36 (33.3)	1.00
Distal	5 (8.8)	8 (15.7)	13 (12.0)	0.38
Ampullary	2 (3.5)	1 (2.0)	3 (2.8)	1.00
Hepatic occupancy of the tumor, *n* (%)				
None	16 (28.1)	5 (9.8)	21 (19.4)	0.027
<50%	32 (56.1)	35 (68.6)	67 (62.0)	0.23
≥50%	9 (15.8)	11 (21.6)	20 (18.5)	0.47
Metastases, *n* (%)				
Lymph node	36 (63.2)	32 (62.8)	68 (63.0)	1.00
Peritoneum	24 (42.1)	18 (35.3)	42 (38.9)	0.55
Lung	15 (26.3)	8 (15.7)	23 (21.3)	0.24
Other^a^	3 (5.3)	1 (2.0)	4 (3.7)	0.62
None	12 (21.1)	14 (27.5)	26 (24.1)	0.50
Primary tumor resection, *n* (%)	12 (21.1)	8 (15.7)	20 (18.5)	0.62
Biliary drainage, *n* (%)				
Total^b^	38 (66.7)	36 (70.6)	74 (68.5)	0.67
With stent	34 (59.7)	34 (66.7)	68 (63.0)	0.55
With surgery	9 (15.8)	7 (13.7)	16 (14.8)	0.79
Type of tumor, *n* (%)				
None^c^	3 (5.3)	18 (35.3)	21 (19.4)	0.00006
ADENO	52 (91.2)	31 (60.8)	83 (76.9)	
ADSQ	1 (1.8)	0	1 (0.9)	
MPC	0	1 (2.0)	1 (0.9)	
SCC	0	1 (2.0)	1 (0.9)	
NEC	1 (1.8)	0	1 (0.9)	
Tumor markers, median (range)				
CEA (ng/mL)	5.5 (0.8–2426)	6.6 (0–226.5)	5.7 (0–2426)	0.76
CA19‐9 (U/mL)	274.2 (3.8–303,749)	496.2 (0–2,197,597)	355.4 (0–2,197,597)	0.45

Abbreviations: ADENO, adenocarcinoma; ADSQ, adenosquamous carcinoma; BSC, best supportive care; MPC, metaplastic carcinoma; NEC, neuroendocrine carcinoma; PS, performance‐status score; SCC, small cell carcinoma.

^a^ Adrenal gland, spleen, bone, and ovary.

^b^ Total of 10 patients (five in the chemotherapy group and five in the BSC group) had stents added postoperatively.

^c^ Includes one case of adenoma.

Comparison of background characteristics between patients grouped according to whether they received chemotherapy or BSC showed that patients in the BSC group were older, had worse ECOG PS, had more tumor lesions in the liver, and included more patients who were histopathologically undiagnosed.

### 3.2 Main causes of death

The main causes of death are shown in Table 2. The most common main cause of death was cholangitis in 33 (30.6%) patients, followed by cachexia in 22 (20.4%) and liver failure in 10 (9.3%) patients. Other main causes associated with tumor progression were seen in 18 (16.7%) cases. In 25 (23.2%) cases, the causes of death were “complications.” The prevalence and distribution of the main causes of death were not significantly different between the chemotherapy and BSC groups.

**TABLE 2:** Main causes of death.

Main cause of death	Chemotherapy group (*N* = 57)	BSC group (*N* = 51)	Total (*N* = 108)	*p*‐Value
Leading causes associated with tumor progression	34 (52.6%)	31 (60.8%)	65 (60.2%)	
Cholangitis	14 (24.5%)	19 (37.3%)	33 (30.6%)	0.21
Cachexia	15 (26.3%)	7 (13.7%)	22 (20.4%)	0.15
Hepatic failure	5 (8.8%)	5 (9.8%)	10 (9.3%)	0.75
Other causes associated with tumor progression in cases without liver failure, cholangitis and cachexia	11 (19.3%)	7 (13.7%)	18 (16.7%)	
DIC (due to tumor invasion)	1 (1.8%)	4 (7.8%)	5 (4.6%)	0.19
Lymphangitis carcinomatosa	3 (5.3%)	0	3 (2.8%)	0.25
Liver abscess	1 (1.8%)	2 (3.9%)	3 (2.8%)	0.60
Pleural dissemination	2 (3.5%)	0	2 (1.9%)	0.50
Gastrointestinal hemorrhage associated with tumor invasion	1 (1.8%)	1 (2.0%)	2 (1.9%)	1.00
Perforation associated with tumor invasion	1 (1.8%)	0	1 (0.9%)	1.00
Meningeal dissemination	1 (1.8%)	0	1 (0.9%)	1.00
Cerebral metastasis	1 (1.8%)	0	1 (0.9%)	1.00
Complications	12 (21.1%)	13 (25.5%)	25 (23.2%)	
Gastrointestinal hemorrhage	0	3 (5.9%)	3 (2.8%)	0.10
Renal failure	2 (3.5%)	2 (3.9%)	4 (3.7%)	1.00
Respiratory failure	2 (3.5%)	1 (2.0%)	3 (2.8%)	1.00
Aspiration pneumonia	0	3 (5.9%)	3 (2.8%)	0.10
Arrhythmia	2 (3.5%)	0	2 (1.9%)	0.50
Cerebral infarction	2 (3.5%)	0	2 (1.9%)	0.50
Thrombosis	2 (3.5%)	0	2 (1.9%)	0.50
Cardiac insufficiency	0	1 (2.0%)	1 (0.9%)	0.47
Decompensated chronic heart failure	0	1 (2.0%)	1 (0.9%)	0.47
Suicide	1 (1.8%)	0	1 (0.9%)	1.00
Complications of procedures^a^	1 (1.8%)	2 (3.9%)	3 (2.8%)	0.60

*Note*: There were no significant differences between the two groups, as assessed by Fisher's exact test.

Abbreviation: DIC, disseminated intravascular coagulation.

^a^ Perforation of the duodenum due to stent placement, respiratory failure due to pleural effusion after pneumothorax treatment, and transfusion‐related acute lung injury.

In patients who were not diagnosed with cachexia or cholangitis as the main cause of death, 28 patients (40.6%) had cachexia, and 36 (52.2%) had cholangitis, among whom 18 (26.1%) had both cachexia and cholangitis (Table S1). Of the patients whose main cause of death was cachexia, 10 patients were diagnosed with “suspected cachexia.”

The background characteristics of patients stratified according to the main cause of death showed significantly more patients with liver masses >50% and significantly fewer patients with peritoneal dissemination among those whose main cause of death was liver failure (Table 3).

**TABLE 3:** Patient background characteristics: classified according to the main cause of death.

	Cachexia **(**N = 22)	Cholangitis **(**N = 33)	Liver failure **(**N = 10)	Other causes^a^ **(**N = 18)	Complications **(**N = 25)	*p*‐ Value
Age (years)						
<59	3 (13.6)	4 (12.1)	1 (10.0)	2 (11.1)	2 (8.0)	0.96
≥60	19 (86.4)	29 (87.9)	9 (90.0)	16 (88.9)	23 (92.0)	
Sex						
Women	6 (27.3)	11 (33.3)	3 (30.0)	4 (22.2)	6 (24.0)	0.92
Men	16 (72.7)	22 (66.7)	7 (70.0)	14 (77.8)	19 (76.0)	
Primary tumor site						
Intrahepatic	5 (22.7)	7 (21.2)	4 (40.0)	5 (27.8)	8 (32.0)	0.73
Perihilar	4 (18.2)	12 (36.4)	3 (30.0)	3 (16.7)	5 (20.0)	0.45
Gall bladder	7 (31.8)	11 (33.3)	2 (20.0)	7 (38.9)	9 (36.0)	0.91
Distal	5 (22.7)	3 (9.1%)	1 (10.0)	2 (11.1)	2 (8.0)	0.59
Ampullary	1 (4.6)	0	0	1 (5.6)	1 (4.0)	0.65
Hepatic occupancy of the tumor						
None	1 (4.6)	0	0	2 (11.1)	1 (4.0)	0.28
<50%	15 (68.2)	16 (48.5)	2 (20.0)	9 (50.0)	17 (68.0)	0.070
≥50%	6 (27.3)	17 (51.5)	8 (80.0)	7 (38.9)	7 (28.0)	0.022
Metastases						
Lymph node	15 (68.2)	23 (69.7)	6 (60.0)	14 (77.8)	18 (72.0)	0.89
Peritoneum	13 (59.1)	14 (42.4)	1 (10.0)	11 (61.1)	13 (52.0)	0.014
Lung	3 (13.6)	9 (27.3)	4 (40.0)	7 (38.9)	5 (20.0)	0.29
Bone	3 (13.6)	2 (6.1)	1 (10.0)	3 (16.7)	4 (16.0)	0.76
Other^b^	3 (13.6)	2 (6.1)	0	4 (22.2)	5 (20.0)	0.06
None	6 (27.3)	7 (21.2)	3 (30.0)	3 (16.7)	4 (16.0)	0.93
Ascites						
None	1 (4.6)	3 (9.1)	0	4 (22.2)	4 (16.0)	0.33
Mild	8 (36.4)	6 (18.2)	4 (40.0)	4 (22.2)	8 (32.0)	0.44
Moderate	7 (31.8)	15 (45.5)	3 (30.0)	7 (38.9)	6 (24.0)	0.54
Massive	6 (27.3)	9 (27.3)	3 (30.0)	3 (16.7)	7 (28.0)	0.92
Pleural fluid						
None	6 (27.3)	11 (33.3)	6 (60.0)	6 (33.3)	7 (28.0)	0.46
Mild	7 (31.8)	12 (36.4)	3 (30.0)	7 (38.9)	8 (32.0)	0.98
Moderate	5 (22.7)	9 (27.3)	1 (10.0)	4 (22.2)	6 (24.0)	0.91
Massive	4 (18.2)	1 (3.0)	0	1 (5.6)	4 (16.0)	0.20
Chemotherapy						
None (BSC)	7 (31.8)	19 (57.6)	5 (50.0)	7 (38.9)	13 (52.0)	0.37
First line						
GC	7 (31.8)	7 (21.2)	3 (30.0)	6 (33.3)	6 (24.0)	0.84
GS	1 (4.6)	0	1 (10.0)	3 (16.7)	1 (4.0)	0.078
GEM	6 (27.3)	6 (18.2)	0	2 (11.1)	5 (20.0)	0.42
S‐1	1 (4.6)	0	0	0	0	0.46
CDDP+CTP‐11	0	1 (3.0)	0	0	0	1.00
TAI	0	0	1 (10.0)	0	**0**	0.093
Primary tumor resection	4 (18.2)	6 (18.2)	2 (20.0)	4 (22.2)	4 (16.0)	0.99

*Note*: The numbers in parentheses represent percentages. The most recent images were evaluated.

Abbreviations: BSC, best supportive care; CDDP + CTP‐11, cisplatin + Irinotecan; GC, Gemcitabine + Cisplatin; GEM, Gemcitabine; GS, Gemcitabine + S‐1; TAI, Transhepatic arterial infusion.

^a^ Other causes associated with tumor progression, excluding liver failure, cholangitis and cachexia.

^b^ Adrenal gland, spleen, soft tissue, muscle, pleura, brain, meninges, thyroid gland, and ovary.

The primary tumor site had no correlation with the main cause of death in this study (Table S2).

### 3.3 Survival

**FIGURE 3:**
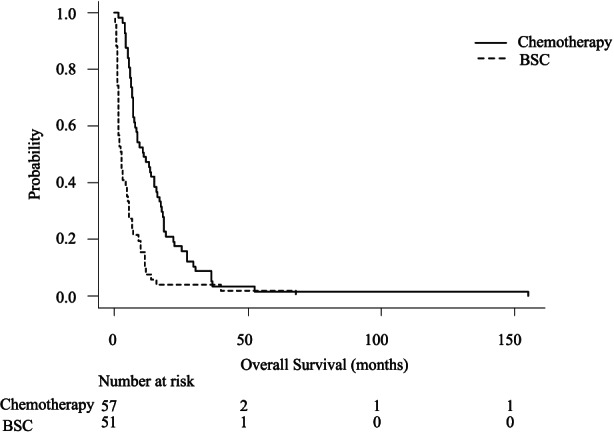
Kaplan–Meier curves showing overall survival (OS) for patients on best supportive care (BSC) versus patients on chemotherapy. Median OS was 334.0 days in the chemotherapy group and 75.0 days in the BSC group.

**FIGURE 4:**
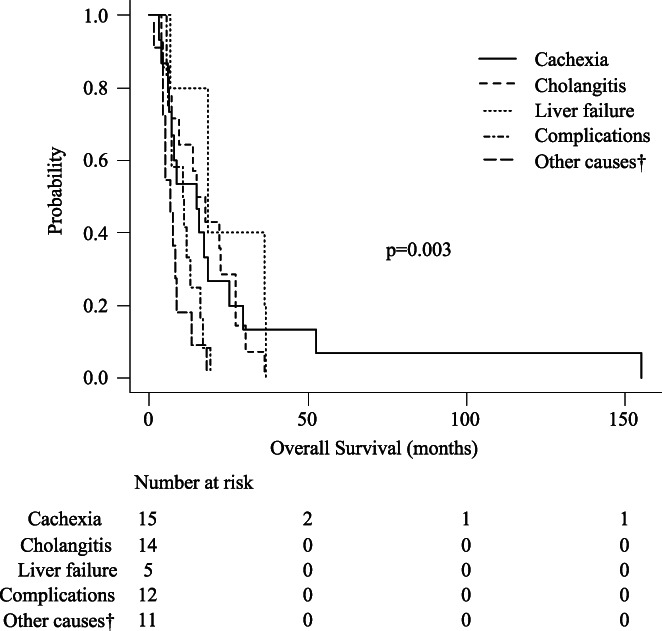
Kaplan–Meier curves showing overall survival (OS) in the chemotherapy group according to cause of death. OS was 453.0 days for cachexia, 499.0 days for cholangitis, 567.0 days for liver failure, 205.0 days for other causes with tumor progression, and 327.5 days for complications in the chemotherapy group (*p* = 0.003).^†^Other causes associated with tumor progression in patients without liver failure, cholangitis, and cachexia.

**FIGURE 5:**
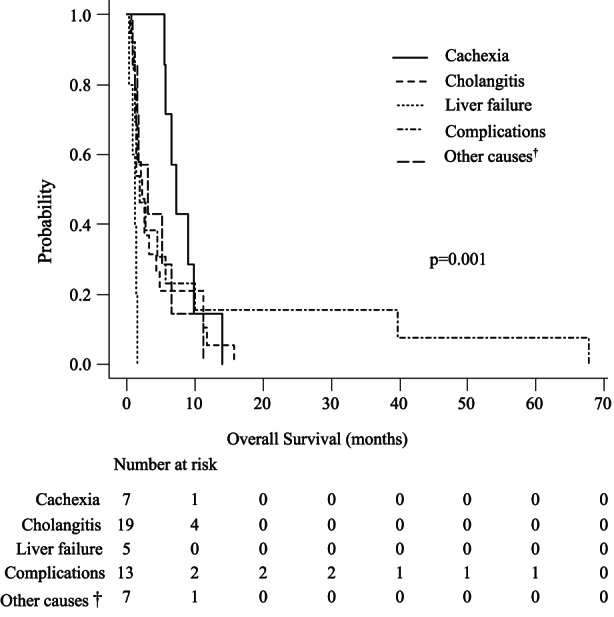
Kaplan–Meier curves showing overall survival (OS) in the best supportive care (BSC) group according to cause of death. OS was 219.0 days for cachexia, 69.0 days for cholangitis, 34.0 days for liver failure, 93.0 days for other causes with tumor progression, and 56.0 days for complications in the BSC group (*p* = 0.014).^†^Other causes associated with tumor progression in patients without liver failure, cholangitis, and cachexia.

The curve of overall survival (OS), defined as the interval between the date of cancer diagnosis and date of death, is shown in Figure 3. Median OS was 334.0 days in the chemotherapy group and 75.0 days in the BSC group. Survival analyzed by main cause of death showed significant differences in both chemotherapy and BSC groups: OS was 453.0 days for cachexia, 499.0 days for cholangitis, 567.0 days for liver failure, 205.0 days for other causes with tumor progression, and 327.5 days for complications in the chemotherapy group (*p* = 0.003), and 219.0 days for cachexia, 69.0 days for cholangitis, 34.0 days for liver failure, 93.0 days for other causes with tumor progression, and 56.0 days for complications in the BSC group (*p* = 0.014) (Figures 4 and 5). In multivariate analysis, patients in the chemotherapy group, especially those with other causes with tumor progression as the main cause of death, had shorter survival times than those with cholangitis as the main cause of death (*p* = 0.043) (Table S3). In multivariate analysis in the BSC group, patients with liver failure as the main cause of death had shorter survival times than those with cachexia and cholangitis as the main causes of death (*p* = 0.003, 0.013) (Table S4).

## 4 DISCUSSION

Shahid et al. previously stated that the poor prognosis of cholangiocarcinoma patients is due to cachexia, liver failure, and cholangitis, although no detailed data were provided.^9^ In this study, we evaluated the main cause of death in patients with unresectable BTC. Determining a single main cause of death in BTC patients is sometimes difficult, because they often have other diseases or conditions complicating their terminal stages. We defined liver failure as the likely primary cause, followed by cholangitis and cachexia, to simplify clarification of the main cause of death. The study findings revealed that the main causes of death in BTC patients were cholangitis (30.6%), followed by cachexia (20.4%), liver failure (9.3%), other causes associated with tumor progression, and complications. We also revealed that cholangitis (40.6%) or cachexia (52.2%) is an underlying condition in many BTC patients at the end of life. Besides, the prevalence and distribution of main causes of death were not significantly different between the chemotherapy and BSC groups. This result suggests that the main cause of death might not be affected by whether or not the patient receives chemotherapy. However, since this was a retrospective study, the relationship between cause of death and survival requires cautious interpretation.

A previous study reported that the main cause of death in cancer patients was infection (36%), followed by hemorrhagic/thromboembolic phenomena (18%), with cachexia accounting for only 1%.^7^ In our study, cholangitis was very common, which we believe represents a disease that is specific to BTC, although it might be difficult to accurately diagnose cholangitis according to the diagnostic criteria in some cases. In our study, more patients were diagnosed with cachexia, and it was determined to be the main cause of death in more cases than previously reported. The high incidence of cachexia might be attributed to its more precise definition in recent years. Sun et al. reported that cachexia was diagnosed in 35.9% of advanced cancer patients, and in 50% of liver/cholangiocarcinoma patients.^17^

Median OS in our study was shorter than that previously reported.^3^, ^4^, ^5^, ^18^, ^19^ The relatively short OS was probably due to the inclusion of more patients with worse ECOG PS, older age, and those treated with monotherapy than previously reported.

This study also analyzed survival for each main cause of death. Multivariate analysis showed that patients in the chemotherapy group had an exceptionally significantly shorter OS among patients whose main cause of death was “other causes with tumor progression.” We hypothesized that the prognosis of patients with advanced BTC was shortened when extrahepatic progression was the main cause of death. Additionally, patients whose cause of death was liver failure had the shortest OS in the BSC group, but tended to have a relatively long OS in the chemotherapy group. This result suggests that liver failure is the most advanced condition in the end stage of BTC, so that treating until the development of liver failure might be the ultimate goal for advanced BTC patients.

There are certain limitations to this study. First, as this was a retrospective observational study, the actual number of patients with cachexia was probably much higher than indicated, due to missing body weight values and the fact that muscle mass and fat mass were not measured. Second, this was a single‐center study with a limited number of patients. Since predicting the main cause of death is deemed necessary for deciding treatment strategies, a multicenter prospective study to more accurately identify the main cause of death in BTC is warranted.

In conclusion, the common causes of death in patients with advanced BTC were cholangitis, cachexia, liver failure, other causes associated with tumor progression, and complications. OS for each cause of death differed significantly in the chemotherapy group. Among cholangitis, cachexia, liver failure, other causes associated with tumor progression and the complications of death in BTC, other causes associated with tumor progression shortened survival in the chemotherapy group, while liver failure tended to shorten survival in the BSC group of BTC patients.

## AUTHOR CONTRIBUTIONS

**Kana Kimura‐Seto:** Conceptualization (lead); data curation (lead); formal analysis (lead); investigation (lead); methodology (lead); resources (lead); supervision (equal); validation (lead); visualization (lead); writing – original draft (lead); writing – review and editing (equal). **Yasushi Kojima:** Conceptualization (lead); data curation (lead); formal analysis (equal); investigation (equal); methodology (lead); project administration (lead); resources (lead); supervision (lead); validation (lead); visualization (lead); writing – original draft (equal); writing – review and editing (lead). **Shiori Komori:** Conceptualization (supporting); data curation (supporting); formal analysis (supporting); investigation (supporting); validation (equal); writing – review and editing (equal). **Yuya Hisada:** Conceptualization (supporting); data curation (supporting); formal analysis (supporting); investigation (supporting); validation (equal); writing – review and editing (equal). **Yuki Otake:** Conceptualization (supporting); data curation (supporting); formal analysis (supporting); investigation (supporting); validation (equal); writing – review and editing (equal). **Yuka Yanai:** Conceptualization (supporting); data curation (supporting); formal analysis (supporting); investigation (supporting); validation (equal); writing – review and editing (equal). **Akiko Saito:** Conceptualization (supporting); data curation (supporting); formal analysis (supporting); investigation (supporting); validation (equal); writing – review and editing (equal). **Naoki Akazawa:** Conceptualization (supporting); data curation (supporting); formal analysis (supporting); investigation (supporting); validation (equal); writing – review and editing (equal). **Yasuo Tanaka:** Conceptualization (supporting); data curation (supporting); formal analysis (supporting); investigation (supporting); validation (equal); writing – review and editing (equal). **Chizu Yokoi:** Conceptualization (supporting); data curation (supporting); formal analysis (supporting); investigation (supporting); validation (equal); writing – review and editing (equal). **Mikio Yanase:** Conceptualization (supporting); data curation (supporting); formal analysis (supporting); investigation (supporting); validation (equal); writing – review and editing (equal). **Junichi Akiyama:** Conceptualization (supporting); data curation (supporting); formal analysis (supporting); investigation (supporting); validation (equal); writing – review and editing (equal). **Natsuyo Yamamoto:** Conceptualization (supporting); data curation (supporting); formal analysis (supporting); investigation (supporting); validation (equal); writing – review and editing (equal). **Kazuhiko Yamada:** Conceptualization (supporting); data curation (supporting); formal analysis (supporting); investigation (supporting); supervision (lead); validation (lead); writing – review and editing (lead).

## CONFLICT OF INTEREST STATEMENT

The authors have no conflict of interest to declare

## ETHICS STATEMENT

This research involving human subjects complies with all the relevant national regulations and institutional policies, was performed in accordance with the tenets of the Helsinki Declaration, and has been approved by the authors' institutional review board or equivalent committee.

## Supporting information


Table S1.

Table S2.

Table S3.

Table S4.

## Data Availability

Data sharing is not applicable to this article as no new data were created or analyzed in this study.
