# Supplementary material for: Main causes of death in advanced biliary tract cancer
Source: Cancer Med. 2023 Mar 29;12(9):10889–98. doi: 10.1002/cam4.5794 (PMC10225207; doi:10.1002/cam4.5794)
Supplement: Supplementary file 1 — Table S1. [file CAM4-12-10889-s001.docx]

Supplementary Table 1. Number of patients who were diagnosed with cachexia and/or cholangitis in patients who were not diagnosed with cachexia or cholangitis as the main cause of death

| Diagnosis of Cachexia and/or Cholangitis  Main cause of death | Cachexia  N=28 (52.8%) | Cholangitis  N=36 (67.9%) | Cachexia and Cholangitis  N=18 (34.0%) | Total  N=53 |
| --- | --- | --- | --- | --- |
| Causes associated with tumor progression | **15 (53.6%)** | **21 (75.0%)** | **9 (32.1%)** | **28** |
| Hepatic failure | 5 (41.7%) | 9 (90.0%) | 5 (41.7%) | 10 |
| DIC (due to tumor invasion) | 3 (60.0%) | 4 (80.0%) | 2 (40.0%) | 5 |
| Lymphangitis carcinomatosa | 3 (100%) | 1 (33.3%) | 1 (33.3%) | 3 |
| Liver abscess | 1 (33.3%) | 3 (100%) | 1 (33.3%) | 3 |
| Pleural dissemination | 1 (50.0%) | 0 (0%) | 0 (0%) | 2 |
| Gastrointestinal hemorrhage associated with tumor invasion | 0 (0%) | 1 (50%) | 0 (0%) | 2 |
| Perforation associated with tumor invasion | 1 (100%) | 0 (0%) | 0 (0%) | 1 |
| Meningeal dissemination | 1 (100%) | 0 (0%) | 0 (0%) | 1 |
| Cerebral metastasis | 0 (0%) | 1 (100%) | 0 (0%) | 1 |
| Complications | **13 (52.0%)** | **15 60.0(%)** | **9 (36.0%)** | **25** |
| Gastrointestinal hemorrhage | 1 (33.3%) | 3 (100%) | 1 (33.3%) | 3 |
| Renal failure | 2 (50.0%) | 2 (50.0%) | 2 (50.0%) | 4 |
| Respiratory failure | 2 (66.7%) | 2 (66.7%) | 1 (33.3%) | 3 |
| Aspiration pneumonia | 1 (33.3%) | 1 (33.3%) | 1 (33.3%) | 3 |
| Arrhythmia | 1 (50.0%) | 2 (100%) | 1 (50.0%) | 2 |
| Cerebral infarction | 2 (100%) | 1 (50%) | 1 (50.0%) | 2 |
| Thrombosis | 1 (50%) | 2 (100%) | 1 (50%) | 2 |
| Cardiac insufficiency | 0 (0%) | 1 (100%) | 0 (0%) | 1 |
| Decompensated chronic heart failure | 1 (100%) | 0 (0%) | 0 (0%) | 1 |
| Suicide | 1 (100%) | 0 (0%) | 0 (0%) | 1 |
| Complications of procesure† | 1 (33.3%) | 1 (33.3%) | 1 (33.3%) | 3 |

DIC: disseminated intravascular coagulation

†Perforation of the duodenum due to stent placement, Respiratory failure due to pleural effusion after pneumothorax treatment, and Transfusion-related acute lung injury
