# Supplementary material for: Main causes of death in advanced biliary tract cancer
Source: Cancer Med. 2023 Mar 29;12(9):10889–98. doi: 10.1002/cam4.5794 (PMC10225207; doi:10.1002/cam4.5794)
Supplement: Supplementary file 2 — Table S2. [file CAM4-12-10889-s003.docx]

Supplementary Table 2. Main cause of death classified by primary tumor site

|  | Intrahepatic  N=29 | Perihilar  N=27 | Gall bladder  N=36 | Distal  N=13 | Ampullary  N=3 | Total  N=108 |
| --- | --- | --- | --- | --- | --- | --- |
| Cholangitis | 7 (24.1%) | 12 (44.4%) | 11 (30.6%) | 3 (23.1%) | 0 (0%) | 33 |
| Cachexia | 5 (17.2%) | 4 (14.8%) | 7 (19.4%) | 5 (38.5%) | 1 (33.3%) | 22 |
| Liver failure | 4 (13.9%) | 3 (11.1%) | 3 (8.4%) | 1 (7.7%) | 0 (0%) | 10 |
| Other causes associated with tumor progression without Liver failure, Cholangitis, and Cachexia | **5 (17.2%)** | **3 (11.1%)** | **7 (19.4%)** | **2 (15.4%)** | **1 (33.3%)** | **18** |
| DIC (due to tumor invasion) | 1 (3.4%) | 0 (0%) | 3 (8.3%) | 1 (7.7%) | 0 (0%) | 5 |
| Lymphangitis carcinomatosa | 0 (0%) | 1 (3.7%) | 2 (5.6%) | 0 (0%) | 0 (0%) | 3 |
| Liver abscess | 1 (3.4%) | 2 (7.4%) | 0 (0%) | 0 (0%) | 0 (0%) | 3 |
| Pleural dissemination | 1 (3.4%) | 0 (0%) | 0 (0%) | 0 (0%) | 1 (33.3%) | 2 |
| Gastrointestinal hemorrhage from tumor invasion | 0 (0%) | 0 (0%) | 1 (2.8%) | 1 (7.7%) | 0 (0%) | 2 |
| Perforation from tumor invasion | 1 (3.4%) | 0 (0%) | 0 (0%) | 0 (0%) | 0 (0%) | 1 |
| Leptomeningeal carcinomatosis | 0 (0%) | 0 (0%) | 1 (2.8%) | 0 (0%) | 0 (0%) | 1 |
| Cerebral metastasis | 1 (3.4%) | 0 (0%) | 0 (0%) | 0 (0%) | 0 (0%) | 1 |
| Complications | **8 (27.6%)** | **5 (18.5%)** | **9 (25.0%)** | **2 (15.4%)** | **1 (33.3%)** | **25** |
| Gastrointestinal hemorrhage | 0 (0%) | 2 (7.4%) | 1 (2.8%) | 0 (0%) | 0 (0%) | 3 |
| Renal failure | 1 (3.4%) | 1 (3.7%) | 2 (5.6%) | 0 (0%) | 0 (0%) | 4 |
| Respiratory failure | 2 (6.9%) | 1 (3.7%) | 0 (0%) | 0 (0%) | 0 (0%) | 3 |
| Aspiration pneumonia | 0 (0%) | 1 (3.7%) | 1 (2.8%) | 0 (0%) | 1 (33.3%) | 3 |
| Arrhythmia | 2 (6.9%) | 0 (0%) | 0 (0%) | 0 (0%) | 0 (0%) | 2 |
| Cerebral infarction | 1 (3.4%) | 0 (0%) | 1 (2.8%) | 0 (0%) | 0 (0%) | 2 |
| Thrombosis | 0 (0%) | 0 (0%) | 2 (5.5%) | 0 (0%) | 0 (0%) | 2 |
| Cardiac insufficiency | 0 (0%) | 0 (0%) | 0 (0%) | 1 (7.7%) | 0 (0%) | 1 |
| Decompensated chronic heart failure | 0 (0%) | 0 (0%) | 0 (0%) | 1 (7.7%) | 0 (0%) | 1 |
| Suicide | 0 (0%) | 0 (0%) | 1 (2.8%) | 0 (0%) | 0 (0%) | 1 |
| Complications of procedures† | 2 (6.9%) | 0 (0%) | 1 (2.8%) | 0 (0%) | 0 (0%) | 3 |

DIC: disseminated intravascular coagulation

†Perforation of the duodenum due to stent placement, Respiratory failure due to pleural effusion after pneumothorax treatment, and Transfusion-related acute lung injury
