# Supplementary material for: Main causes of death in advanced biliary tract cancer
Source: Cancer Med. 2023 Mar 29;12(9):10889–98. doi: 10.1002/cam4.5794 (PMC10225207; doi:10.1002/cam4.5794)
Supplement: Supplementary file 4 — Table S4. [file CAM4-12-10889-s002.docx]

Supplementary Table 4. Multivariate analysis of patients in the best supportive care (BSC) group

|  | Cachexia | Cholangitis | Liver failure | Complications |
| --- | --- | --- | --- | --- |
| Cholangitis | 1.00 | - | - | - |
| Liver failure | 0.003 | 0.013 | - | - |
| Complications | 1.00 | 1.00 | 0.22 | - |
| Other causes | 1.00 | 1.00 | 0.056 | 1.00 |

P value adjustment method: Bonferroni correction

†Other causes associated with tumor progression without Liver failure, Cholangitis, and Cachexia.
